# Pollen and spore monitoring in the world

**DOI:** 10.1186/s13601-018-0197-8

**Published:** 2018-04-04

**Authors:** J. T. M. Buters, C. Antunes, A. Galveias, K. C. Bergmann, M. Thibaudon, C. Galán, C. Schmidt-Weber, J. Oteros

**Affiliations:** 1 Center of Allergy and Environment (ZAUM), Member of the German Center for Lung Research (DZL), Technische Universität München/Helmholtz Center, Biedersteinerstrasse 29, 80802 Munich, Germany; 2 ICAAM – Institute of Mediterranean Crop and Environmental Sciences, University of Évora, Évora, Portugal; 3 Allergy-Center-Charité, Charité University Hospital, Berlin, Germany; 4 RNSA (Réseau National de Surveillance Aérobiologique), Brussieu, France; 5 Department of Botany, Ecology and Plant Physiology, University of Córdoba, International Campus of Excellence on Agrifood (ceiA3), Córdoba, Spain

**Keywords:** Allergy, Pollen, Moulds, Spores, Monitoring, Trap, Hirst, Durham, Cour, Automatic, Stations, Map

## Abstract

**Background:**

Ambient air quality monitoring is a governmental duty that is widely carried out in order to detect non-biological (“chemical”) components in ambient air, such as particles of < 10 µm (PM_10_, PM_2.5_), ozone, sulphur dioxide, and nitrogen oxides. These monitoring networks are publicly funded and air quality data are open to the public. The situation for biological particles that have detrimental effects on health, as is the case of pollen and fungal spores, is however very different. Most pollen and spore monitoring networks are not publicly funded and data are not freely available. The information regarding which biological particle is being monitored, where and by whom, is consequently often not known, even by aerobiologists themselves. This is a considerable problem, as local pollen data are an important tool for the prevention of allergic symptoms.

**Objective:**

The aim of this study was to review pollen monitoring stations throughout the world and to create an interactive visualization of their distribution.

**Methods:**

The method employed to collect information was based on: (a) a review of the recent and historical bibliography related to pollen and fungal spore monitoring, and (b) personal surveys of the managers of national and regional monitoring networks. The interactive application was developed using the R programming language.

**Results:**

We have created an inventory of the active pollen and spore monitoring stations in the world. There are at least 879 active pollen monitoring stations in the world, most of which are in Europe (> 500). The prevalent monitoring method is based on the Hirst principle (> 600 stations). The inventory is visualised as an interactive and on-line map. It can be searched, its appearance can be adjusted to the users’ needs and it is updated regularly, as new stations or changes to those that already exist can be submitted online.

**Conclusions:**

The map shows the current situation of pollen and spore monitoring and facilitates collaboration among those individuals who are interested in pollen and spore counts. It might also help to improve the monitoring of biological particles up to the current level employed for non-biological components.

## Background

Pollen and fungal spores have detrimental effects on health [1–7] and it is therefore logical that these airborne allergenic particles are monitored throughout the world. Monitoring the air quality of non-biological components in ambient air is commonplace and is undertaken world-wide, mostly on the basis of legal exposure limits. The airborne components such as PM_10_, PM_2.5_, SO_2_, NO_X_ and O_3_ that are monitored, often with standardized methods, may be different depending on the country that is carrying out the monitoring. The fact that this monitoring is carried out with public financing signifies that most of the data from these networks are available to the public at no cost and are often published with open access on the internet. Citizens can, therefore, easily assess the quality of the air that they breathe with a minimal lag time thanks to these online networks.

The situation of biological particles is, however, completely different. Only few countries like MeteoSwiss (Switzerland) and RNSA (France) have state-owned monitoring networks. Biological particles, including pollen and fungal spores (spores), were first monitored with medical purposes in 1870 by Blackley in the UK [8], and the oldest continuous pollen record dates back to 1943 in Cardiff, the UK, a station that has used a Hirst pollen trap since 1954 [9–11]. The volumetric Hirst-type pollen and spore trap is still one of the instruments most widely used for pollen and spore monitoring [12]. Records for non-biological components are more recent: for example, the CO_2_ concentrations in ambient air at Mauna Loa, Hawaii, date back to 1958 [13]. Another difference between biological and non-biological air quality monitoring is that non-biological particles are collected by law as particles of < 10 µm or smaller (PM_10_, PM_2.5_), whereas biological particles are often > 10 µm [14–16]. Some pollen, such as *Urticamembranaceae* Poir (Urticaceae) [17], and some fungal spores are < 10 µm in diameter (e.g. *Aspergillus* or *Penicillium* [18]), but none are smaller than fine particles of < 2.5 µm. Non-biological air quality parameters are, therefore, either gasses or small particles, while biological air quality parameters are predominately very large particles.

Despite the large size of pollen, the imperfect capacity of common PM_10_ samplers to separate particles by size [19] signifies that up to 15% of birch (21 µm, range approx. 15–27 µm) or grass pollen (25–45 µm) falls into the PM_10_ fraction (particles of between 2.5 and 10 µm) [15, 20, 21], and the situation is similar for other pollen and moulds. The reason why very large particles are found in < 10 µm fractions of air is that the instruments used to collect particulate matter from ambient air do not completely separate them. These instruments, which are mostly impactors, have less efficient separation characteristics then their names suggest: PM_10_ implies that particles > 10 µm are separated from the smaller particles. This is not the case, and approximately 15% of pollen, and even pollen which is larger than 20 µm, can be detected in the fraction of air that should contain only particles of < 10 µm. Pollen and fungal spores are, therefore, collected with the existing air-quality monitoring networks, but very poorly and these technical limitations consequently signify that networks monitoring non-biological components are not useful for monitoring biological particles.

About 30% of the population suffers from some type of allergy to airborne pollen [6], which may have extreme effects on their health, including death [22]. However, few governments own pollen monitoring stations or run a monitoring network for pollen and/or fungal spores. Most pollen and spore networks are privately owned and the data they produce are not freely available.

For allergy sufferers, the location of pollen monitoring stations is unclear and mostly unknown: many Apps deliver pollen forecasts, but it is unclear how the data are obtained and are of little interest to the users, as they assume that sufficient data support the pollen flight prognosis. This is often not the case. For instance, Bavaria in Germany has 12 million inhabitants but only 3 non-public pollen monitoring stations are operating (www.pollenstiftung.de), despite the availability of many Apps. The quality of the pollen flight prognosis is consequently, questionable. Finding the location of pollen monitoring stations could, therefore, be useful for stakeholders. The creation of a map of pollen monitoring stations will improve access to local pollen data and will, hopefully, benefit those with allergies, the medical profession and, of course, aerobiologists. Our aim was, therefore, to a review pollen and spore monitoring stations throughout the world and to develop a practical visualisation method to disseminate their data.

## Methods

The inventory of active pollen monitoring stations was created by: (1) reviewing the relevant bibliography and (2) contacting authors or the network managers of pollen networks. Information on pollen monitoring stations was obtained by phone, email or post. A small questionnaire was sent, which requested information on what was monitored, since when, at which location, using which collection method (Hirst-type, brand type Burkard or Lanzoni, automatic or another sampling system), the availability of quality control, and the station owner’s most recent contact data. This questionnaire is also accessible in an interactive format in the map application. The questionnaire makes it possible to fill in a.csv database which serves as input data for the map.

The map was programmed in “R” [23]. Maps are instantaneously displayed at https://www.zaum-online.de/pollen-map.html. Stations that are currently running are displayed by default, but historic sites can also be made visible. New information for the map (changes of owner, a new station etc.) can be submitted to either the App-administrator or the map itself online via the “contact form (Modify info, add station, contact)” button. The information is then reviewed by the App-administrator and, if correct, entered in the database, after which the new information is displayed. It is not possible to make a modification without the App-administrator, thus preventing the misuse of the map.

## Results and discussion

**Fig. 1:**
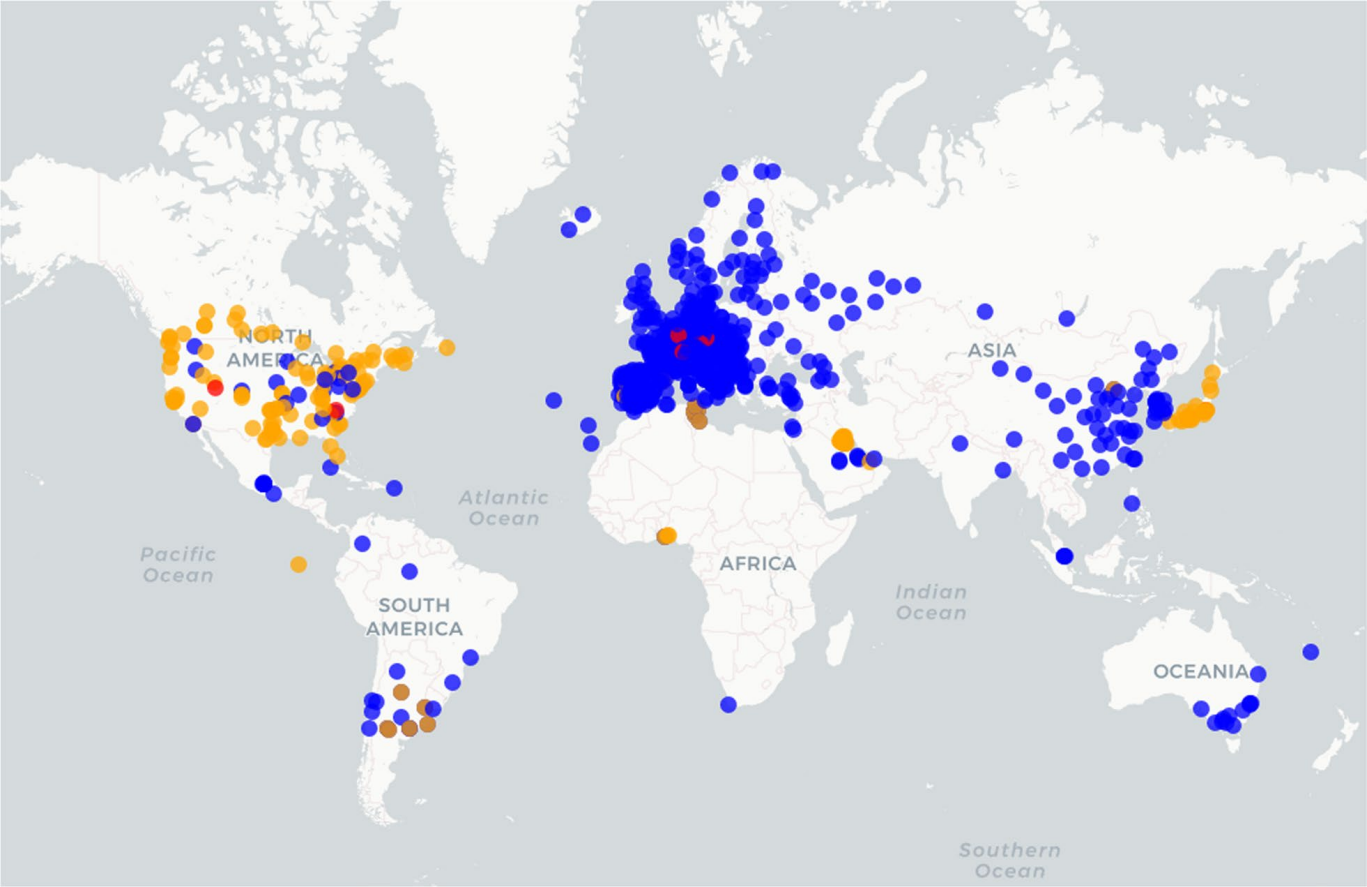
Screen shot of the interactive map of pollen and fungal spore monitoring stations in the world. The map is web-based, zoomable down to street level, shows the contact information of each station, and can be searched and displayed according the users’ needs. The map is constantly updated and is available at: https://www.zaum-online.de/pollen-map.html; http://www.eaaci.org/patients/resources; https://oteros.shinyapps.io/pollen_map (accessed 25-01-2018). Blue dots (Hirst trap), red (Automatic station), orange (other manual)

In order to clarify who is monitoring which biological particle and where, we created an up-dated inventory. We subsequently constructed a map of the pollen monitoring sites throughout the world. We received feed-back from 1011 sites. Because our old map [24] was already outdated upon publishing, we opted for an interactive on-line map. This map can be accessed at: https://www.zaum-online.de/pollen-map.html; http://www.eaaci.org/patients/resources/; https://oteros.shinyapps.io/pollen_map/ (see Fig. 1). Other websites also show information on pollen monitoring networks in the world (http://www.worldallergy.org/pollen/, accessed Jan 2018), but most information is incomplete or is limited to one country (https://www.uco.es/rea/infor_rea/estaciones.htm, accessed Jan 2018), or shows only links to certain national websites (https://www.polleninfo.org/country-choose.html, accessed Jan 2018). Access to this map was made open to the public in June 2017, and has already exceeded 250 h/month. The map is constantly updated and individuals who have, or know of, a station that is not currently on the map may use the contact or submission buttons to make that station visible to all. The map does not show any pollen or spore data, only where a station is located (zoom down to street level), what is monitored (pollen, spores or both) and who to contact in order to obtain information regarding that station (or pollen date). The map allows its users to search for specific stations, and also has a cluster view and filtering options for the monitoring features.

The map makes access to pollen and spore information freely available. As pollen and spores do not stop at borders, but measuring networks and their dissemination activities do, it was more complicated to obtain data from stations abroad in the past, simply because access was complicated. The new map only shows the contact information of all the data providers that agreed to be in. The map will foster international cooperation and enable policy makers to compare their local situation with that of the rest of the world. Patients who suffer from hay fever and members of the medical profession who are treating hay fever patients will profit by finding the most suitable monitoring station from which to obtain pollen or spore data for comparison with the patient’s symptoms. This could improve diagnosis.

Our review accounts for at least 879 open running pollen traps in the world from a total of 1020 records (Fig. 1). As can be observed, most of the traps are distributed in the northern hemisphere, the majority in Europe. The continent with the weakest coverage is Africa. The top six countries by number of active pollen stations are: Japan (143), Italy (88), USA (85), France (85), Spain (77) and Germany (44). The Environmental Agency of Japan owns the biggest network (120 automatic traps), while RNSA of France owns the biggest Hirst-based network (84). The inventory focused on currently operational stations. Some cities are densely monitored by several pollen traps e.g. Milan, Seoul, Tokyo, Toronto, Sydney, Madrid, Mexico DC or Paris. Discontinued historical locations were not specifically inventoried, although interested owners can include their stations on the map. Of those stations that are still open, 70% are based on the Hirst method (Table 1) [12]. Hirst is the prevalent method on all continents with the exception of America, where only 28% of the traps are Hirst and most are based on Rotorod technology. We observed a 50–50% equilibrium as regards the abundance of the two main Hirst trap brands: Burkard (http://www.burkard.co.uk/, http://www.burkardscientific.co.uk) and Lanzoni (www.lanzoni.it).

**Table 1:** Pollen and mould monitoring stations in the world

	Automatic	Hirst	Other	
Africa	0	6	3	9
America	2	43	106	151
Asia	120	38	24	182
Europe	8	517	0	525
Oceania	0	12	0	12
	130	616	133	879

Only information regarding those stations that were active in 2016 (879 stations) from the total inventory (1020 stations) is displayed

Japan is the pioneer in automatic monitoring, with 120 stations based on the KH technology [25]. Automatic monitoring in Japan is possible because the spectrum of pollen whose identification is of interest is very limited [26]. According to Pollen-Sense (http://pollensense.com/), there are only two automatic stations in the whole of the American continent. Europe is reported to have 8 automatic stations: 2 based on Plair PA-300 Rapid E [27] and 6 based on BAA-500 [28]. However, automation is increasing: a network is being built in Switzerland and a network of 8 BAA-500 is being built in Bavaria (Germany). The time resolution provided by automatic stations is between 1 and 3 h. Of the manual methods, Hirst allows the minimal time resolution of 2 h, which is provided by 50% of the stations. The other stations provide average concentrations of 24 h. About 50% of those stations that are open also monitor fungal spores in ambient air; the fungal species monitored depend on the country, but almost always include *Alternaria* spp. and often *Cladosporium* spp. Further statistics can be obtained from the online map site.

## Conclusions

The majority of active monitoring stations are based on the Hirst principle (616 of 879 stations), but technological developments now allow automatic monitoring.

This map makes it clear where and by whom pollen and fungal spores are monitored in the world. It is similar to the publicly available maps created for non-biological (chemical) pollutants and makes it clear that, in our opinion, biological particle monitoring is a neglected aspect of air quality monitoring. We trust that the map will address this discrepancy.
